# Asymmetric triplex metallohelices stabilise DNA G-quadruplexes in promoter oncogene sequences and efficiently reduce their expression in cancer cells

**DOI:** 10.1080/14756366.2023.2198678

**Published:** 2023-04-05

**Authors:** Jaroslav Malina, Hana Kostrhunova, Hualong Song, Peter Scott, Viktor Brabec

**Affiliations:** a Czech Academy of Sciences, Institute of Biophysics, Brno, Czech Republic; b Department of Chemistry, University of Warwick, Coventry, UK

**Keywords:** Metallohelices, G-quadruplexes, telomeres, expression of oncogenes, DNA synthesis

## Abstract

Some metallo-supramolecular helical assemblies with size, shape, charge and amphipathic architectures similar to short cationic α-helical peptides have been shown to target and stabilise DNA G-quadruplexes (G4s) *in vitro* and downregulate the expression of G4-regulated genes in human cells. To expand the library of metallohelical structures that can act as efficient DNA G4 binders and downregulate genes containing G4-forming sequences in their promoter regions, we investigated the interaction of the two enantiomeric pairs of asymmetric Fe(II) triplex metallohelices with a series of five different DNA G4s formed by the human telomeric sequence (*hTelo*) and in the promoter regions of *c-MYC, c-KIT*, and *k-RAS* oncogenes. The metallohelices display preferential binding to G4s over duplex DNA in all investigated G4-forming sequences and induced arrest of DNA polymerase on template strands containing G4-forming sequences. Moreover, the investigated metallohelices suppressed the expression of *c-MYC* and *k-RAS* genes at mRNA and protein levels in HCT116 human cancer cells, as revealed by RT-qPCR analysis and western blotting.

## Introduction

In addition to the canonical double helix, DNA can also fold into alternative higher-order structures that are believed to participate in controlling essential genetic processes such as transcription, replication, and telomere maintenance^1–3^ and are involved in the development of many human pathologies^4^. DNA G-quadruplexes (G4s) formed by guanine-rich sequences that assemble into G-quartets stabilised by Hoogsteen hydrogen bonds and monovalent cations^5^ are highly stable tetra-stranded structures, and their formation has been confirmed in human telomeres and several key genome regions; available evidence suggests that their stabilisation downregulates gene expression^6^. Hence G-quadruplexes have attracted growing attention as possible drug targets also because they are predominantly formed in promoters of oncogenes, such as *c-MYC*^7^*, c-KIT*^8^*, k-RAS*^9^*, BCL2*^10^, or *VEGF*^11^. Remarkably, the *c-MYC* and *k-RAS* oncogenes are overexpressed in 70%^12^ and 30% of all human cancers, respectively, and as it has been demonstrated, small-molecule stabilization^13^ of G4s located in their promoter regions downregulated *c-MYC*^14^ and *k-RAS*^13^ expression. The *c-KIT* oncogene, as another example, is implicated in the progression of several types of cancer, mainly gastrointestinal stromal tumours^15^. It contains in its promoter region two G4-forming sequences; stabilisation of G-quadruplexes formed in these sequences by appropriate ligands inhibits the expression of *c-KIT*^16^.

Considering the role played by G4s in cancer pathogenesis, it is understandable that much effort has been invested into the development of G4-interacting small molecules, including metal complexes^17^^,^^18^. Typical synthetic G4 binders contain flat aromatic chromophores for π-π stacking with G-quartets, positively charged side chains for interactions with loops and grooves of the G4, and steric bulk preventing intercalation between DNA base pairs^5^^,^^19^. This is in contrast to natural proteins that interact with G4s^20^ via α-helical recognition units^21^. For example, the helicase DHX36 interacts with the top quartette of a DNA G4 via the hydrophobic region of a cationic α-helix with the addition hydrogen-bonding to the adjacent DNA backbone^22^.

**Figure 1.**
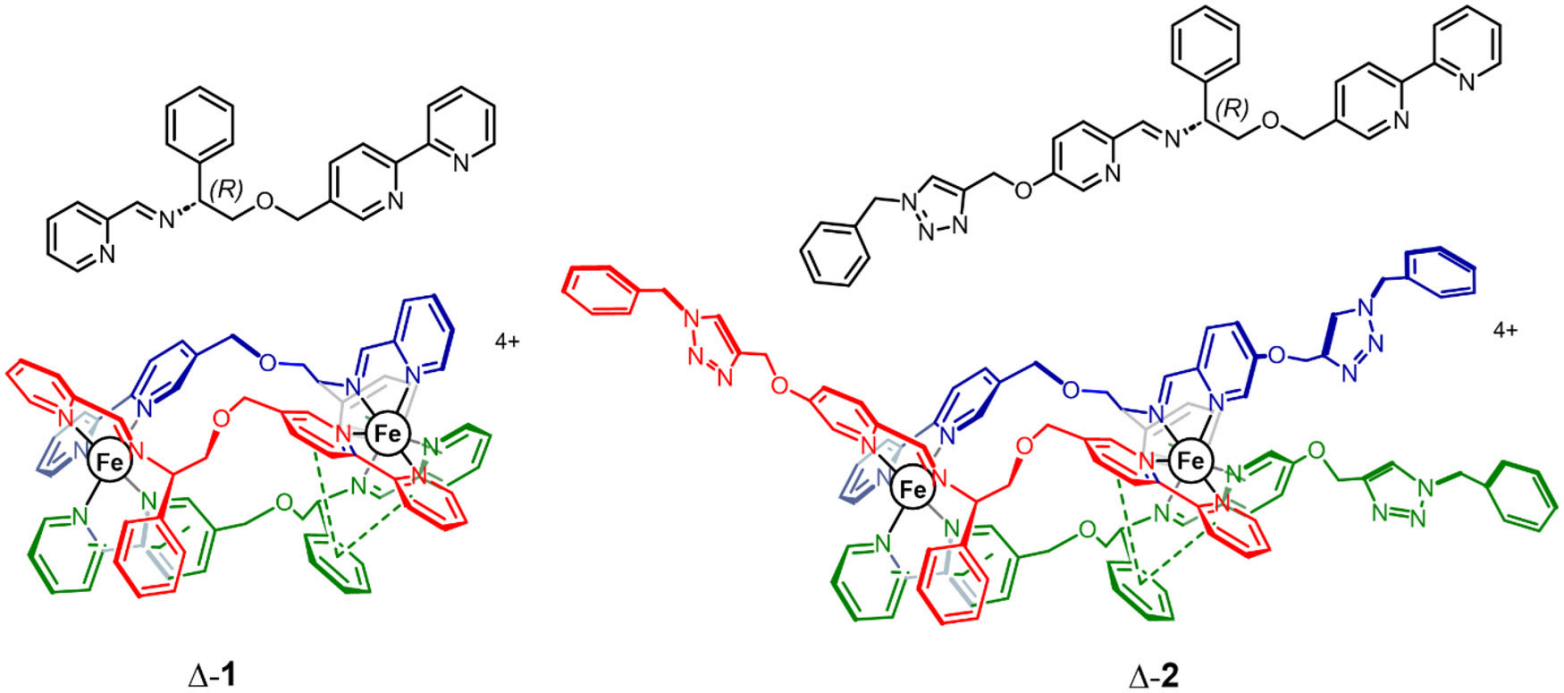
Triplex metallohelices used in this study. Each assembly comprises three ligand strands L, colourised separately and shown inset in black for clarity. The *R*_c_,Δ_Fe_,HHT-[Fe_2_L_3_]^4+^ enantiomers shown are denoted **Δ-1** and **Δ-2**. The mirror images *S*_c_,Λ_Fe_,HHT-[Fe_2_L_3_]^4+^ are denoted **Λ-1** and **Λ-2** in the text.

We have recently reported that some metallo-supramolecular helical assemblies with size, shape and charge similar to short cationic α-helical peptides^23^^,^^24^ can target and stabilise G4s *in vitro* and downregulate the expression of G4-regulated genes in human cells^25^^,^^26^. While α-helices are inherently asymmetric, being formed from directional oligopeptide strands, the above-mentioned chiral metallohelical structures are of high symmetry due to synthetic feasibility and the use of symmetric strands AB-BA. Recently, we developed a new strategy for the preparation of asymmetric architectures such as **1** and **2** (Figure 1) where directional strands AB-CD self-assembly with high selectivity into optically pure head-to-head-to-tail (HHT) systems in the absence of head-to-head-to-head (HHH) isomers^27^^,^^28^. The outcome is a short α-helix-like architecture, highly resistant to unfolding, with an amphipathic architecture comprising a relatively hydrophobic face (lower red/green strand regions in Figure1) dominated by inter-strand π-π stacks which shield the charge arising from the metal, and an upper more hydrophilic face (red/blue strands) where the metal coordination sphere is more exposed, and which can be decorated by hydrogen-bonding units such as the triazoles in **2**.

In tests of 14 compounds against a panel of seven cancer and four non-cancer cell lines of different tissue origins, such metallohelices were shown to easily outperform cisplatin in terms of activity and selectivity. In particular, the benzyltriazole enantiomers **2** display an array of advantages over the parent compounds **1** including higher potency and selectivity, suppression of metastatic capacity and cancer stem cell targeting^28^.

For these reasons we selected the two enantiomer pairs of asymmetric triplex metallohelices **1** and **2** shown in Figure 1 for an investigation of their interaction with a series of five DNA G4s (*hTelo*, *c-myc*, *c-kit1*, *c-kit2, k-ras*) that are formed by the human telomeric sequence (*hTelo*) and in the promoter regions of *c-MYC, c-KIT*, and *k-RAS* oncogenes by using fluorescence intercalator displacement (FID) assays, fluorescence resonance energy transfer (FRET) melting assays, and DNA polymerase stop assays. The RT-qPCR and western blotting were used to explore the downstream effects of **1** and **2** in human colon cancer cells HCT116. Both metallohelices are extremely stable in aqueous media with respect to unfolding i.e. hydrolytic dissociation from the Fe centres, even in the presence of strong acid (pH 1)^28^.

## Results and discussion

### Fluorescence indicator displacement (FID) assay

**Figure 2.**
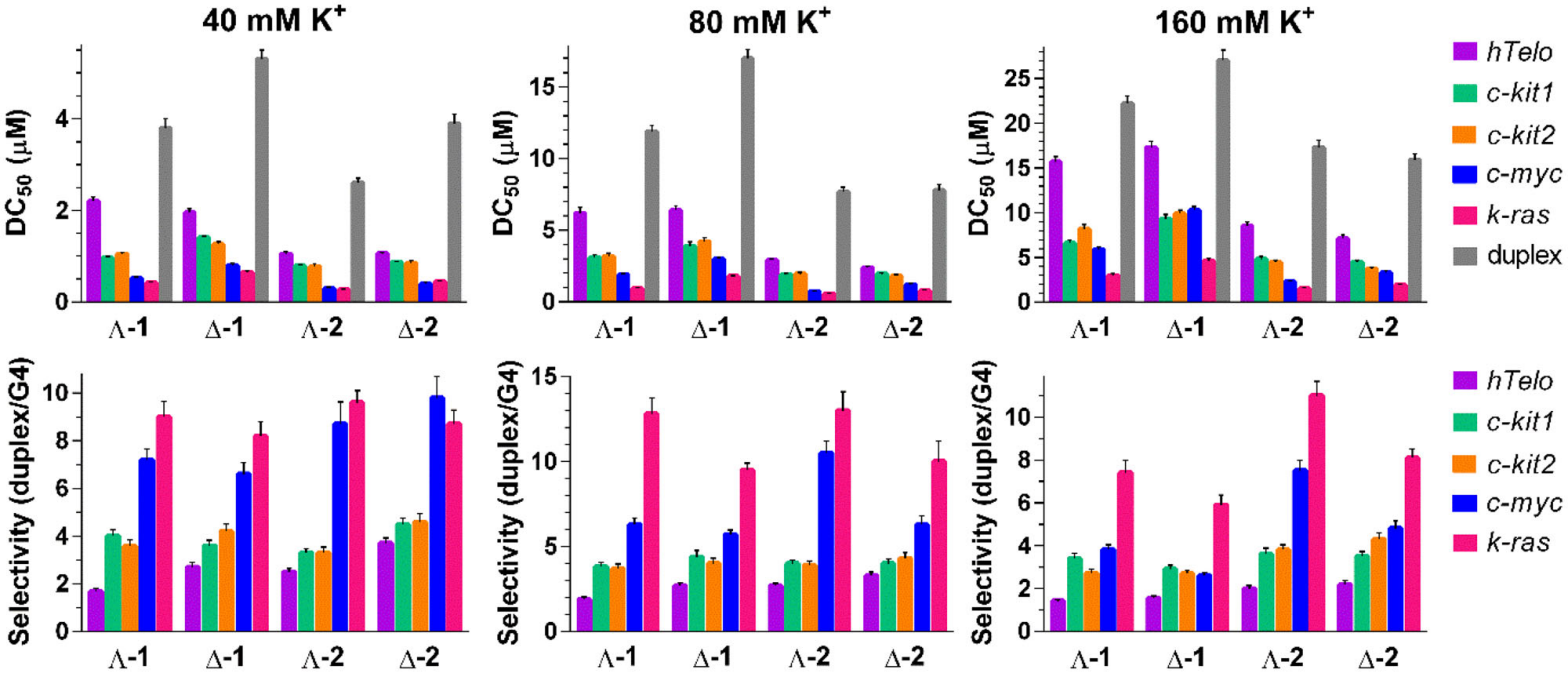
DC_50_ values [μM] (upper panels) for DNA G4s and DNA duplex determined by FID upon the addition of metallohelices in 10 mM potassium phosphate buffer (pH 7) and increasing concentrations of KCl (total concentrations of K^+^ are indicated in the Figure 2). The results are expressed as mean ± SD from 2 independent experiments. Selectivity indexes (lower panels) of metallohelices towards DNA G4s in 10 mM potassium phosphate buffer (pH 7) and increasing concentrations of KCl. The values of the binding selectivity for each metallohelix were calculated as a ratio between the DC_50_ values obtained for the duplex and G4 DNA.

We used FID assay^29^ for establishing binding affinities and selectivities of **1** and **2** towards a short duplex DNA (26_ds) and *hTelo, c-kit1*, *c-kit2*, *c-m*yc, and *k-ras* G4 DNA. Selected G4s differ by their topology and length, and nucleotide composition of intervening loops. While the human telomeric G4 (*hTelo*) adopts a basket-type antiparallel arrangement^30^, *c-myc*^31^*, c-kit1*, *c-kit2*^32^, and *k-ras*^33^ fold into the parallel topology. The DNA duplex and folded G4s (0.25 µM) were mixed with 0.5 µM thiazole orange (TO) and titrated with metallohelices while the fluorescence of TO was monitored. To compare the binding affinities of individual metallohelices, the DC_50_ values corresponding to the concentration of the metallohelix causing a 50% reduction of TO fluorescence (see plots in Figure S1) were determined. The obtained DC_50_ values are graphically depicted in bar graphs in Figure 2, and the numerical values are presented in Tables S1, S3, and S5. Measurements were conducted in three different concentrations of K^+^ (40, 80 and 160 mM) to investigate the importance of electrostatic interactions for the binding of metallohelices to G4 and duplex DNA. Inspection of the data reveals that **1** and **2** can indeed displace TO more efficiently from G4 than from duplex DNA. Generally, metallohelices exhibited low TO displacing potency towards *hTelo* while maintaining high activity against *k-ras* and *c-myc* G4s throughout the entire K^+^ concentration range. The lowest DC_50_ values between 0.27 and 0.45 µM were recorded for the displacement of TO from *k-ras* and *c-myc* G4s by **Λ-2** and **Δ-2** in 40 mM K^+^. **Λ-1** was slightly less effective with DC_50_ values of 0.42 and 0.53 μM, respectively. Such low DC_50_ values (≤0.5 µM) indicate that both enantiomers of **2** and **Λ-1** have excellent affinity for *k-ras* and *c-myc* G4s^29^.

We have recently reported that the binding affinities of metallohelices to G4 and duplex DNA depend on the ionic strength and that as the ionic strength increases the interaction of metallohelices with duplex DNA is generally weakened more than that with G4 DNA^25^^,^^26^. This effect is manifested by an enhancement of the selectivity indexes (SIs) calculated as the ratio of DC_50_ values for duplex and G4 DNA. As can be seen in Figure 2 (see also Tables S2, S4 and S6), the SI values of the Λ- and Δ-enantiomers of **1** and **2** are not steadily elevated with increasing concentration of K^+^ but follow different trends for different G4s. Unlike previously studied symmetric metallohelices, we did not observe severalfold increments of SIs upon adding K^+^^26^. Instead, changes in SIs were small and occurred in both directions depending on the metallohelix and G4 structure. Doubling the concentration of K^+^ from 40 to 80 mM resulted in an enhancement of the binding selectivities of all metallohelices towards *k-ras* G4, and at the same time, **Λ-2** was the only compound whose binding selectivity towards all G4s raised. When the concentration of K^+^ was further increased to 160 mM, all SIs were lowered; most of them were even lower than at 40 mM K^+^. The highest SI values of 12.8 and 13 were recorded for the binding of **Λ-1** and **Λ-2** towards *k-ras* G4 at 80 mM K^+^, respectively, but generally, good binding selectivity of both metallohelices towards *k-ras* and *c-myc* G4s was observed with SIs starting from 9.5 and 5.7, respectively. These values are lower than those recorded for some of the previously studied symmetric metallohelices^26^, but on the other hand, they are higher than SIs of Ni(II) cylinders^25^ that were still able to downregulate the expression of the *c-MYC* oncogene in human cells. Data also demonstrate that **2** is a stronger and more selective binder to *k-ras* and *c-myc* G4s than **1**.

### FRET melting assays

**Figure 3.**
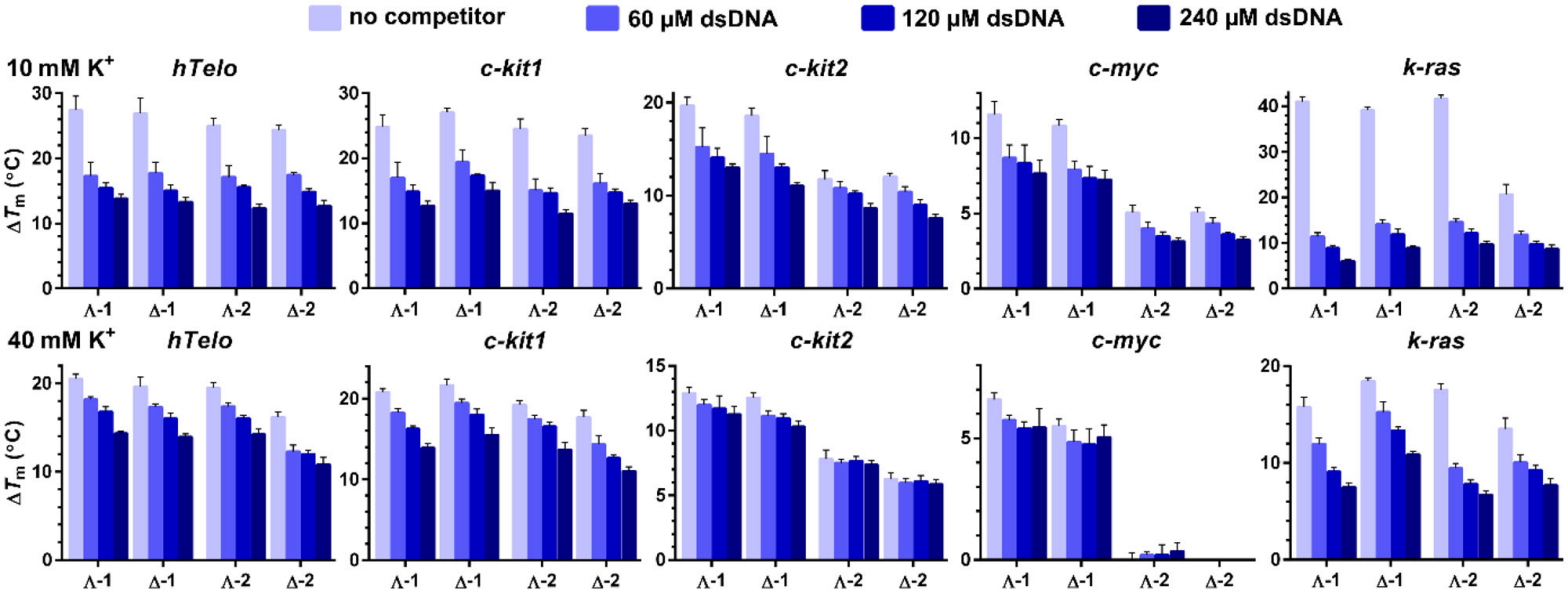
Δ*T*_m_ values for the fluorescently labelled *hTelo*, *c-kit1*, *c-kit2*, *c-myc*, and *k-ras* DNA G4s (0.4 μM) determined by FRET upon addition of 0.8 μM enantiomers of **1** and **2** in the presence of increasing concentrations of dsDNA (indicated in Figure 3). The measurements were carried out in 10 mM potassium phosphate (pH 7) (upper panels) and 10 mM potassium phosphate and 30 mM KCl (pH 7) (lower panels). The results are expressed as mean ± SD from three independent experiments.

FRET melting assay^34^ was employed to assess the ability of **1** and **2** to stabilise DNA G4 structures. In addition, the use of fluorescently labelled oligonucleotides in combination with competing unlabelled double-stranded (ds) DNA allows evaluation of the binding selectivity of metallohelices towards G4s. Representative melting curves for DNA G4s mixed with **2** in the presence of various concentrations (60–240 µM, concentration per nucleotide) of dsDNA are shown in Figures S2 and S3. Since the thermal stabilities of some G4s are quite high, the measurements were carried out in just 10 mM potassium phosphate buffer to keep the melting temperatures (*T*_m_s) low. The *T*_m_s of *c-myc*, *c-kit1*, *c-kit2*, *hTelo*, and *k-ras* in 10 mM K^+^ were 77.6, 51.0, 68.5, 52.3 and 42.3 °C, respectively. The calculated Δ*T*_m_ values are presented in column plots in Figure 3, and their inspection reveals that **1** and **2** can exert some stabilising effect against G4s even in the presence of a large excess of dsDNA, which is in accordance with the preferential binding of metallohelices to G4 over dsDNA observed in the FID assays. The binding selectivity of metallohelices was further corroborated by measuring the thermal stability of a short DNA duplex (26_ds) mixed with **1** and **2**. The melting curves in Figure S4 show that metallohelices increased the *T*_m_ of duplex DNA by less than 1 °C and that this stabilising effect vanished in the presence of just 60 µM dsDNA.

A closer inspection of the data in Figure 3 reveals some inconsistency between results from the FRET melting and FID assays. For instance, the differences between stabilising activities of Λ- and Δ-enantiomers were minimal apart from *k-ras* G4, where **Δ-2** was substantially less effective than remaining metallohelices, but the most conspicuous is the reduction of stabilising activity of metallohelices towards *k-ras* G4 in the presence of competing dsDNA although FID assays produced for this G4 structure the highest SIs. It must be taken into account that FID assays were conducted at ≥40 mM K^+^ and that the DNA binding properties of metallohelices are dependent on the ionic strength. Hence, we repeated FRET melting experiments at 40 mM K^+^. The *T*_m_s of *c-myc*, *c-kit1*, *c-kit2*, *hTelo*, and *k-ras* in these conditions raised to 87.4, 58.5, 77.4, 61.2 and 49.0 °C, respectively. The results summarised in lower panels in Figure 3 and Table 1 (see also melting curves in Figures S2 and S3) show that Δ*T*_m_s in the absence of dsDNA were markedly lower, while the values of Δ*T*_m_ in the presence of 240 µM dsDNA were just slightly affected. As a result, the loss of stabilising activity of metallohelices in the presence of 240 µM dsDNA in 40 mM K^+^ was much smaller than in 10 mM K^+^, which indicates increased binding selectivities of metallohelices to G4s at 40 mM K^+^. The % of stabilisation loss in the presence of 240 µM dsDNA in 10 *vs.* 40 mM K^+^ was calculated for all G4s to quantify the impact of higher K^+^ concentration on the binding selectivity of **1** and **2**, and the results are presented in Table 1. The addition of dsDNA had a less negative effect on G4 stabilisation at the higher (40 mM) K^+^ concentration. For instance, **1** and **2** lost between ∼85 and 57% of their capacity to stabilise *k-ras* in 10 mM K^+^ in the presence of 240 µM dsDNA, and these values were lowered to ∼62–41% in 40 mM K^+^. Acquired data, however, do not allow a direct comparison of the thermal stabilisation capacities of **1** and **2** among different G4s because the *T*_m_s of the tested G4s are spread over a wide interval of temperatures.

**Table 1.** Loss of the stabilising ability of metallohelices (0.8 µM) towards DNA G4s (0.4 µM) in the presence of 240 µM dsDNA in 10 and 40 mM K^+^.

Metallohelix	Δ*T*_m_ (°C) in the absence of dsDNA in 10/40 mM K^+^	Δ*T*_m_ (°C) in the presence of 240 µM dsDNA in 10/40 mM K^+^	% of stabilisation loss in 10/40 mM K^+^
*hTelo*			
**Λ-1**	27.6/20.5	14.0/14.5	49.3/29.3
**Δ-1**	27.1/19.8	13.5/14.1	50.2/28.8
**Λ-2**	25.2/19.6	12.5/14.4	50.4/26.5
**Δ-2**	24.6/16.4	12.9/11.0	47.6/32.9
*c-kit1*			
**Λ-1**	25.0/21.0	12.9/14.0	48.4/33.3
**Δ-1**	27.2/21.8	15.2/15.7	44.1/28.0
**Λ-2**	24.8/19.4	11.7/13.9	52.8/28.4
**Δ-2**	23.7/17.8	13.2/11.3	44.3/33.5
*c-kit2*			
**Λ-1**	19.8/13.0	13.2/11.3	33.3/13.1
**Δ-1**	18.7/12.7	11.1/10.4	40.6/18.1
**Λ-2**	11.9/7.9	8.7/7.4	26.9/6.3
**Δ-2**	12.1/6.4	7.6/6.0	37.2/6.2
*c-myc*			
**Λ-1**	11.7/6.6	7.8/5.5	33.3/16.7
**Δ-1**	10.9 / 5.5	7.3/5.1	33.0/7.2
**Λ-2**	5.2/n.a.	3.2/n.a.	38.5/n.a.
**Δ-2**	5.2/n.a.	3.3/n.a.	36.5/n.a.
*k-ras*			
**Λ-1**	41.3/15.9	6.3/7.7	84.7/51.6
**Δ-1**	39.3/18.6	9.3/11.0	76.3/40.9
**Λ-2**	41.8/17.7	10.0/6.8	76.1/61.6
**Δ-2**	21.0/13.7	9.0/7.9	57.1/42.3

In the case of the most stable G4s *c-myc* and *c-kit2*, with the *T*_m_s values of 77.6 and 68.5 °C in 10 mM K^+^ and 87.4 and 77.4 °C in 40 mM K^+^, respectively, it can be observed that **2** provided lower stabilisation than **1** which is not in agreement with the results from the FID assays and for *c-myc* in 40 mM K^+^ both enantiomers of **2** completely lost their capacity to stabilise this structure. The inconsistencies between the results obtained by FID assays and FRET melting assays when assessing the binding affinity of metallo-supramolecular helices towards DNA G4s were already observed in our previous publications^25^^,^^26^. The discrepancy may result from different experimental conditions but also from different binding modes of the ligands. Typical G4 binders are planar molecules that stack on the top or bottom G-quartet of the G4 and displace TO by direct competition, whereas compounds susceptible to establishing additional interactions with loops or grooves, such as metallohelices might displace TO by both direct and indirect competition if they bind to a nearby site. So, when comparing results obtained by the FID and FRET melting assays, it should be considered that these two methods differ in the relative weights of the π-stacking and the electrostatic contributions.

FRET melting and FID assay are established and widely used methods for studying the interactions of ligands with G4s. However, the results obtained by both methods may be affected by some of their shortcomings and that is why we used in this work both methods at the same time. We compared the results from both methods and, in addition, we tried, based on the suggestion of one reviewer, to confirm these results also using circular dichroism (CD) melting experiments testing the stabilisation of the *k-ras* G4 by metallohelices **1** and **2** (Figures S5–S7). In the case of metallohelices **1** and **2**, the use of CD melting experiments is complicated by the fact that the enantiomers themselves show a strong CD signal in the region where DNA absorbs (Figure S5). Figure S6 shows that *k-ras* G4 itself rapidly denatures when the temperature is raised above 30 °C (see also Figure S7), while in the presence of both enantiomers **1** and **2**, its temperature stability is significantly higher (Figure S7). Although the results are not identical to those from FRET melting, they confirm the conclusions of our study made on the basis of FRET melting experiments and FID assay, i.e. that **1** and **2** significantly increase the stability of G4s.

### Taq DNA polymerase stop assay

**Figure 4.**
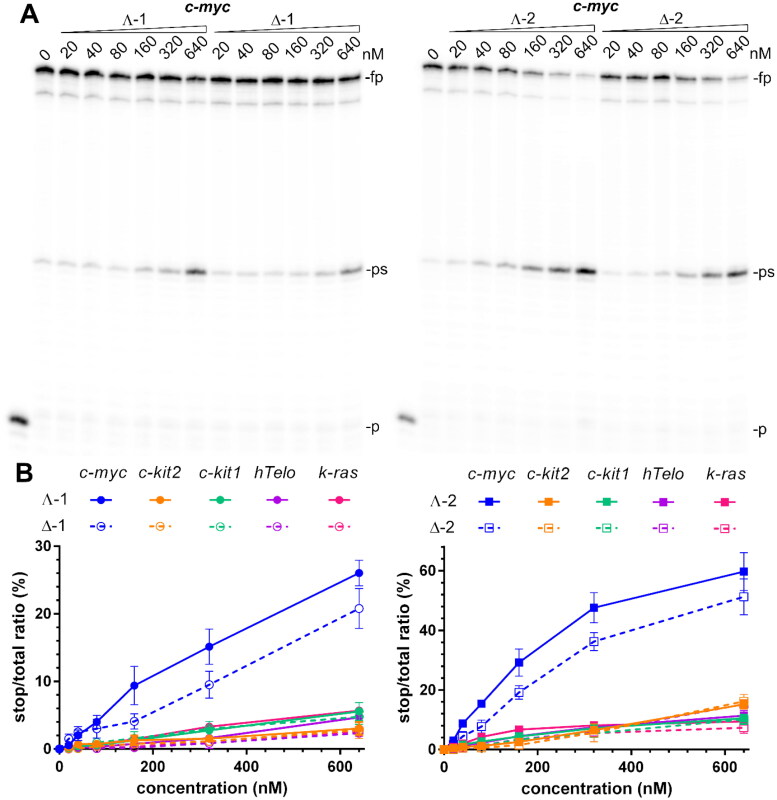
Inhibition of Taq polymerase DNA synthesis on templates (30 nM) with *c-myc*, *c-kit2*, *c-kit1*, *hTelo*, and *k-ras* G4-forming sequences in the presence of increasing concentrations of metallohelices. A. Autoradiograms of 12% PAA sequencing gels with products of DNA synthesis on the template containing *c-myc* G4-forming sequence in the presence of increasing concentrations of the Λ- and Δ-enantiomers of **1** and **2**. *fp*, *ps*, and *p* correspond to full-length product, pausing site by G4, and primer, respectively. B. Plots showing the ratio of the radiation corresponding to pausing sites to total radiation of the lane vs. the concentration of **1** and **2** enantiomers. The results are expressed as mean ± SD from two independent experiments.

A DNA polymerase stop assay is frequently used for screening for G4-binders capable of inducing a polymerase arrest by stabilising an intramolecular G4 structure in the template strand^35^. Gels in Figures 4(A), S8, and S9 show the products of *Taq* DNA polymerase primer elongation reactions on DNA templates containing *c-myc*, *hTelo*, *c-kit1*, *c-kit2*, and *k-ras* G4-forming sequences in the presence of increasing concentrations (0–640 nM) of **1** and **2**. In the absence of metallohelices, there was only slight pausing of the *Taq* DNA polymerase when it bypasses the G-rich site on the template DNA. However, upon adding **1** and **2**, enhanced pausing is observed at the same site as in the absence of metallohelices. This suggests that the stabilisation of G4 structures by **1** and **2** is responsible for the premature termination of DNA synthesis. To demonstrate that the polymerase arrest results from the stabilisation of G4 by metallohelices and not from their interaction with the DNA polymerase or primer/template constructs, additional experiments were performed with modified *c-myc* and *c-kit2* templates containing mutated sequences that were unable to fold into G4 structures because the middle guanines in runs of three guanines participating in the formation of G4s were replaced by cytosines. Gels in Figure S10 confirm that DNA polymerisation on these mutated templates was not prematurely terminated in the presence of 640 nM metallohelices and therefore, the stabilisation of G4s in the template strands by **1** and **2** is responsible for the arrest of DNA synthesis. Gels, including those in Figures 4(A), S8, and S9, were analysed, and data quantified as the percentage of normalised stop product *vs*. the total intensity per lane and plotted against the concentration of metallohelices (Figures 4(B) and S11). It can be immediately noticed that **2** is more effective in blocking DNA polymerase than **1** and that both metallohelices, particularly their Λ-enantiomers, displayed the highest inhibiting activity towards the template containing *c-myc* G4-forming sequence. This is partly contradictory to the results of FID assays, where both metallohelices exhibited high binding affinity and selectivity towards not only *c-myc* but also towards *k-ras* G4. On the other hand, the higher binding affinity of **2** for all G4s determined by the FID assays complies well with the enhanced potency of **2** to prematurely terminate DNA synthesis (Figures 4(B) and S11).

It should be noted that the result of DNA replication across a G4 depends not only on the stability but also on the topology of the G4 structure. It has been reported that the parallel and antiparallel G4s block DNA polymerase more efficiently than hybrid G4s despite having similar thermodynamic stability^36^. The inhibitory effect of the G-binder is stability-dependent as well as topology-selective but is also dominated by the mode of interaction between the G-binder and G4. Compounds capable of forming additional interactions within the loop regions of the G4 were found to be more potent inhibitors as the interactions with nucleotide bases in the loops reduced the ability of the polymerase to unwind the G4 structure^37^.

Taken together, the results showed that **1** and **2** at submicromolar concentrations can inhibit DNA polymerisation by stabilising G4 structures in the template strands, although they are slightly less potent than some of the previously studied symmetric metallohelices^26^.

### Biological assays

**Figure 5.**
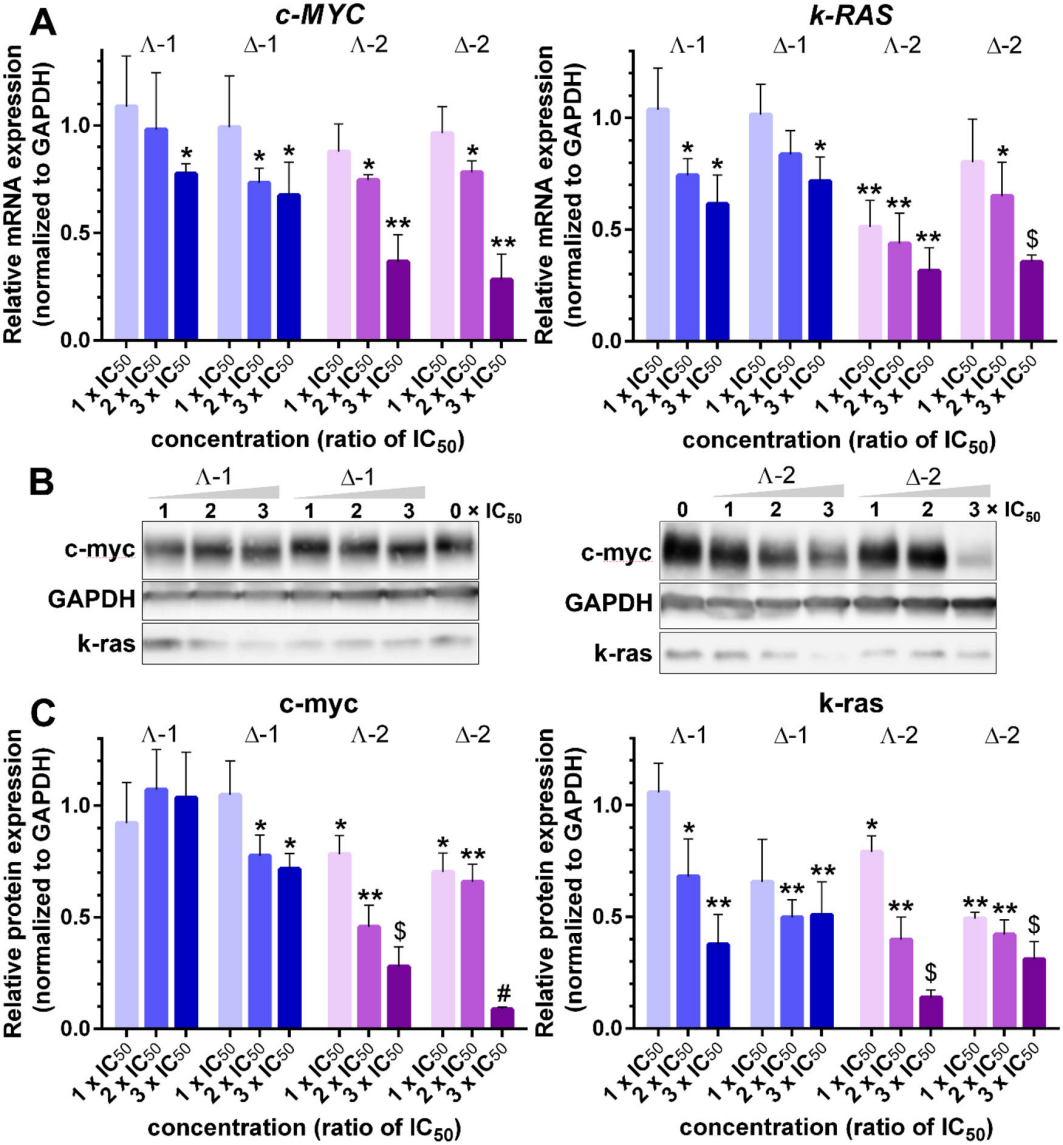
A. Relative expression of *c-MYC* and *k-RAS* mRNA in HCT116 cells treated with Λ- and Δ-enantiomers of **1** and **2** at concentrations corresponding to 1×, 2×, and 3 × IC_50_, respectively, normalised to GAPDH. B. Western blot images of c-myc, k-ras, and GAPDH levels in HCT116 cells treated with increasing concentrations (indicated above gels) of **1** and **2**. C. Relative expression of c-myc and k-ras proteins normalised to GAPDH. Data represent the mean ± SD of two to four independent experiments. The stars indicate a statistically significant difference determined by the Student t-test (*p ≤ .05; **p ≤ .01; $*p* ≤ .001; #*p* ≤ .0001).

The results mentioned above prompted us to explore the impact of **1** and **2** on the expression of *c-MYC* and *k-RAS* oncogenes in human colon cancer cells HCT116. To ensure that the cells were exposed to equitoxic doses of the tested metallohelices, we determined the IC_50_ values in HCT116 cells after a 72-h treatment using an MTT assay. The values of IC_50_ in Table 2 show that both enantiomers of **2** were several-fold more active than those of **1** and that the Λ-enantiomers were more potent than the Δ-enantiomers. These data are in good agreement with the previous findings^28^. In the following experiments, the cells were treated with compound concentrations corresponding to 1×, 2×, and 3× IC_50_ for 24 h, and the expression of *c-MYC* and *k-RAS* oncogenes was followed by RT-qPCR and western blot analysis (Figure 5). The relative fold changes of mRNA and protein expression were calculated by normalising against the constitutively expressed housekeeping gene, glyceraldehyde-3-phosphate dehydrogenase (GAPDH).

**Table 2.** IC_50_ values determined with MTT assay in HCT116 cells after a 72-h treatment.

Metallohelix	IC_50_ (µM)
**Λ-1**	3.9 ± 0.6
**Δ-1**	17 ± 3
**Λ-2**	0.5 ± 0.1
**Δ-2**	1.5 ± 0.3

The bar graphs in Figure 5(A) show that all four metallohelices inhibited *c-MYC* and *k-RAS* mRNA synthesis in a dose-dependent manner and that **2** was more efficient than **1**. Western blot analysis of c-myc and k-ras protein levels (Figure 5(B,C)) reveals a dose-dependent decrease in c-myc and k-ras amounts for **Δ-1**, **Λ-2**, and **Δ-2**, only for **Λ-1** and c-myc the inhibition of protein synthesis was not detected. All four metallohelices were more efficient inhibitors of k-ras protein production, and the inhibiting effect was stronger in **Λ-2**, and **Δ-2** treated cells.

Overall, these results demonstrate that both metallohelices **1** and **2** can downregulate the expression of G4-controlled oncogenes, such as *c-MYC* and *k-RAS*, in human cancer cells.

## Conclusions

We have studied the interaction of two enantiomeric pairs of asymmetric triplex metallohelices with a series of five different DNA G4s formed by the human telomeric sequence (*hTelo*) and in the promoter regions of *c-MYC, c-KIT*, and *k-RAS* oncogenes. The results showed that metallohelices **1** and **2** preferred binding to G4 over duplex DNA and that their binding affinities towards various G4 structures varied. The highest affinities were observed for *k-ras* and *c-myc* G4s with parallel topology, while the weakest binding was recorded for *hTelo* G4, which folds into a basket-type antiparallel arrangement, suggesting that the investigated metallohelices favour binding to parallel-stranded DNA G4s. Noticeably, the Λ-enantiomers of **1** and **2** exhibited markedly higher binding affinities towards *c-myc* and *k-ras* G4s than the Δ-enantiomers, and at the same time, both enantiomers of **2** were more potent displacers of TO from all G4s than their **1** analogs. The binding selectivities of **1** and **2** towards G4s over duplex DNA were dependent on the ionic strength as it was observed for previously studied symmetric metallohelices^26^ and Ni(II) cylinders^25^. The values of selectivity indexes (SIs) raised markedly when the concentration of K^+^ was doubled from 40 to 80 mM and then slightly declined when the concentration of K^+^ was further increased to 160 mM. The SIs of **1** and **2** to *c-myc* and *k-ras* G4s at 80 mM K^+^ reached respectable values between 5.7 and 13. Since cancer cells have a much lower K^+^ concentration (∼60 mM) than normal cells (∼150 mM) due to overexpression of K^+^ channels^38^, the enhanced binding selectivity of **1** and **2** towards G4 DNA in <150 mM K^+^ concentrations might be partly responsible for the observed selective antiproliferative activity of metallohelices against cancer cells. Both metallohelices were capable of stabilising G4 structures in the presence of an excess of competitor dsDNA and inducing the arrest of DNA polymerase on template strands containing G4-forming sequences. Compound **2**, as a stronger binder to G4 DNA, was also more effective in stopping DNA polymerase on all G4-containing templates than **1**. While the details of the binding modes of **1** and **2** remain to be resolved, we note the presence (Figure 1) of the lower relatively hydrophobic region comprised of π-π stacks capable of further supramolecular interactions such as stacking to G-tetrads. Indeed, such intermolecular interactions are present in the crystal structure of **1**^27^. Further, the superior performance of **2** over **1** may be a result of the additional interactions of the hydrogen bond donor benzyl triazole moieties with the phosphate backbone and bases within the grooves and loops of the G4. RT-qPCR analysis and western blotting revealed that **1** and **2** suppressed the expression of *c-MYC* and *k-RAS* genes at mRNA and protein levels in HCT116 human cancer cells in a dose-dependent manner, with **2** being more efficient, although both metallohelices were applied at equitoxic concentrations.

Taken together, these results suggest that the suppressing effect of **1** and **2** on G-regulated oncogenes may contribute to their selective antiproliferative activity against human cancer cell lines. Our findings also add to the growing library of metallohelical structures that can act as efficient DNA G4 binders and downregulate genes containing G4-forming sequences in their promoter regions. Furthermore, the results demonstrate that triplex metallohelices offer a promising platform for the development of high affinity and potentially selective G4-targeted ligands.

## Experimental section

### Chemicals and reagents

Metallohelices (Figure 1) were synthesised as previously published^27^^,^^28^. HPLC-purified synthetic oligonucleotides were purchased from Eurofins Genomics (Ebersberg, Germany) and used with no further purification.

### FRET measurements

The double-labelled (FAM, 6-carboxyfluorescein; TAMRA, 6-carboxytetramethylrhodamine) oligonucleotides F21T_*hTelo*, 5′-FAM-GGGTTAGGGTTAGGGTTAGGG-TAMRA-3′, F21T_*c-kit1*, 5′-FAM-GGGAGGGCGCTGGGAGGAGGG-TAMRA-3′, F20T_*c-kit2*, 5′-FAM-GGGCGGGCGCGAGGGAGGGG-TAMRA-3′, F21T_*c-myc*, 5′-FAM-GAGGGTGGGTAGGGTGGGTAA-TAMRA-3′, F21T_*k-ras*, 5′-FAM-GGGCGGTGTGGGAATAGGGAA-TAMRA-3′, and F26T_ds, 5′-FAM-CAATCGGATCGAATTCGATCCGATTG-TAMRA-3′ were allowed to fold at a 4 µM concentration in 10 mM potassium phosphate buffer (pH 7) by heating to 95 °C for 5 min followed by slow cooling to RT and then put in the refrigerator overnight. Metallohelices (0.8 μM) were added to the mixtures of oligonucleotides (0.4 μM) with various concentrations of double-stranded (ds) DNA from *Micrococcus luteus* (Sigma-Aldrich, Prague, Czech Republic) either in 10 mM potassium phosphate buffer (pH 7) alone or 10 mM potassium phosphate buffer and 30 mM KCl. Samples were prepared in a total volume of 40 µL in 200 µL microtubes. Measurements were carried out on a real-time PCR instrument RotorGene 6000 (Corbett Research), with excitation at 470 ± 10 nm and detection at 510 ± 5 nm. The temperature was increased at a rate of 0.7 °C/min from 26 to 98 °C, and the fluorescence readings were taken at 1 min intervals. The melting temperatures (*T*_m_) were determined within the supplied application software by examining the first derivatives of the melting curves.

### FID measurements

Oligonucleotides 22_*hTelo*, 5′-AGGGTTAGGGTTAGGGTTAGGG-3″,

22_*c-kit1*, 5′-AGGGAGGGCGCTGGGAGGAGGG-3″,

21_*c-kit2*, 5′-CGGGCGGGCGCGAGGGAGGGG-3″,

22_*c-myc*, 5′-TGAGGGTGGGTAGGGTGGGTAA-3″,

22_*k-ras*, 5′-AGGGCGGTGTGGGAATAGGGAA-3′, and

26_ds 5′-CAATCGGATCGAATTCGATCCGATTG-3″ were allowed to fold at a 6.25 µM concentration in 10 mM potassium phosphate buffer (pH 7) and 30, 70 or 150 mM KCl by heating to 95 °C for 5 min followed by slow cooling to RT and then put in the refrigerator overnight. Oligonucleotides (0.25 µM) were mixed with 0.5 µM thiazole orange (Sigma-Aldrich, Prague, Czech Republic) and placed into a 0.5 cm quartz cuvette in a total volume of 0.6 ml. Small volumes (typically 1.2 µL) of metallohelices were added to the mixture to obtain the desired concentration and thoroughly mixed by pipetting. Samples in the cuvette were left to equilibrate for 3 min at 25 °C before data reading was taken. Measurements were carried out using a Varian Cary Eclipse spectrofluorophotometer and the following parameters: 501 and 538 nm as the excitation and emission wavelengths, respectively, 10 nm as the excitation and emission slit widths, and 3s as the averaging time.

### Taq DNA polymerase stop assay

Primer P22, 5′-TAATACGACTCACTATAGCAAT-3′ (20 nM) was 5′-end-labelled with [γ-32]ATP (Hartmann analytic GmbH, Braunschweig, Germany) and T4 polynucleotide kinase (New England Biolabs, MA, USA) according to the standard procedure and then annealed to one of the complementary templates:

*hTelo_*templ, 5′-TCCAACTATGTATACTTAGGGTTAGGGTTAGGGTTAGGG ACATAT CGATGAAATTGCTATAGTGAGTCGTATTA-3′;

*c-myc_*templ, 5′-TCCAACTATGTATACTTTGAGGGTGGGTAGGGTGGGTAAACATAT CGATGAAATTGCTATAGTGAGTCGTATTA-3′;

*c-kit1*_templ, 5′-TCCAACTATGTATACTTAGGGAGGGCGCT GGGAGGAGGGACATA TCGATGAAATTGCTATAGTGAGTCGTATTA-3′;

*c-kit2*_templ,

5′-TCCAACTATGTATACTTCGGGCGGGCGCGAGGGAGGGGACATATCGATGAAAT TGCTATAGTGAGTCGTATTA-3′;

*k-ras*_templ, 5′-TCCAACTATGTATACTTAGGGCGGTGTGGGAATAGGGAAACATAT CGATGAAATTGCTATAGTGAGTCGTATTA-3′;

*c-myc*_templ_ctrl, 5′- TCCAACTATGTATACTTTGAGCGTGCGTAGCGTGCGTAAACA TATCGATGAAATTGCTATAGTGAGTCGTATTA-3′;

*c-kit2*_templ_ctrl, 5′- TCCAACTATGTATACTTCGCGCGCGCGCGAGCGAGCGGACA TATCGATGAAATTGCTATAGTGAGTCGTATTA-3′

(30 nM) in 10 mM Tris-HCl (pH 8) buffer containing 25 mM KCl and 1.5 mM MgCl_2_ by heating to 95 °C for 5 min followed by slow cooling to RT and then put in the refrigerator overnight. Primer-template constructs were mixed with increasing concentrations of metallohelices and left for 10 min at RT. The primer elongation reactions were initiated by adding dNTP (final concentration of 200 μM) and 4 units of *Taq* DNA polymerase (New England Biolabs, Beverly, MA). Samples (final volumes of 10 µL) were incubated for 60 min at 55 °C for *c-myc* and *c-kit2*, 45 °C for *c-kit1*, and 40 °C for *k-ras* and *hTelo* G4 containing templates. The DNA polymerisation was terminated by adding an equal volume of 2 × concentrated formamide loading buffer followed by heating to 90 °C for 3 min. Products of DNA synthesis were separated on a 12% PAA sequencing gel. Gels were exposed to a phosphor imaging plate and scanned using a GE Healthcare FLA 7000 laser scanner.

### Cytotoxic/antiproliferative activity

HCT116 cells were seeded in 96-well plates at a density of 1.5 × 10^3^ cells/well in complete DMEM medium (high glucose 4.5 g/L, 50 µg/mL gentamycin, and 10% heat-inactivated FBS) and grown for 16 h (37 °C, 5% CO_2_, humidified atmosphere). The cells were then treated with metallohelices (0–100 µM) for 72 h. 10 µL MTT [3–(4,5-dimethyl-2-thiazolyl)-2,5-diphenyl-2H-tetrazolium bromide; 2.5 mg mL^−1^] was added to the wells for another 4 h. The medium was removed, and the formazan products were dissolved in 100 µL DMSO. Absorbance was read at 570 nm (620 nm reference) on SPARK multimode plate reader (Tecan). IC_50_ values were calculated as concentrations causing a 50% decrease in the absorbance signal of non-treated control wells. The experiment was performed three times with triplicates in each assay.

### RT-qPCR analysis of c-MYC and k-RAS mRNA expression

HCT116 cells were seeded (60 mm Petri dishes) at a density of 3 × 10^5^ cells/dish and grown overnight. Tested compounds were then added at concentrations corresponding to 1×, 2×, and 3× IC_50_ values (determined in the previous experiment). Following a 24-h incubation, the cells were harvested, washed, and pelleted. RNA was extracted from the pellets using RNasy® Plus Mini Kit (QIAGEN) according to the manufacturer’s instructions. A one-step RT-qPCR assay combining reverse transcription and amplification thermal cycling was used (Luna® universal one-step RT-qPCR; New England Biolabs, MA, USA). The reactions were run on Illumina Eco real-time PCR instrument (Illumina, CA, USA) using the following thermal profiles: 10 min at 55 °C (reverse transcription); 1 min at 95 °C (initial denaturation); 43 cycles of 10 s at 95 °C and 30 s at 60 °C (denaturation and extension, respective). The following primer sequences were used; GAPDH-F, 5′-GTCTCCTCTGACTTCAACAGCG-3″; GAPDH-R, 5′-ACCACCCTGTTGCTGTAGCCAA-3″; C-MYC-F, 5′-CCTGGTGCTCCATGAGGAGAC-3″; C-MYC-R, 5′-CAGACTCTGACCTTTTGCCAGG-3″; K-RAS-F, 5′- TGTTCACAAAGGTTTTGTCTCC −3″; K-RAS-R, 5′- CCTTATAATAGTTTCCATTGCCTTG −3″. Melting curve analysis and template-free negative controls were run to confirm specific single-product amplification. GAPDH was used as the internal control. Relative mRNA expression is shown as fold change (2^−ΔΔCt^)^39^.

### Western blot analysis of c-myc and k-ras protein expression

HCT116 cells were seeded and treated as in the previous experiment, harvested, and the cell pellets were lyzed with ice-cold RIPA buffer (supplemented with proteinase inhibitors as in the manufacturer’s recommendations). The protein extracts were then cleared with centrifugation (16 000 rpm/8 min), mixed with 2 × LBS Buffer (4% SDS; 10% 2-mercaptoethanol; 20% glycerol; 0. 004% bromophenol blue and 0.125 M Tris-HCl) and heated (95 °C/10 min). The proteins were resolved on a 4–15% SDS-PAGE (Mini-PROTEAN® TGX^TM^ Precast Gels), transferred to PVDF membrane, and detected with appropriate antibodies: Anti-GAPDH antibody, mouse monoclonal (Sigma-Aldrich; 1:200), Anti-c-Myc antibody [Y69] ab32072 (Abcam; 1:1000), Anti-K-Ras antibody 703 345 (Invitrogen; 1:5000), Goat anti-Mouse IgG (H + L) Secondary antibody, HRP (ThermoFisher Scientific; 1:200) and Goat Anti-Rabbit IgG H & L (HRP) ab205718 (Abcam; 1:1000). SignalFire^TM^ ECL Reagent (A + B) was used as a substrate, and the luminescence was visualised with Amersham Imager 680. Band densities were evaluated using Aida image analysis software.

## Supplementary Material

Supplemental Material
