# Supplementary material for: Asymmetric triplex metallohelices stabilise DNA G-quadruplexes in promoter oncogene sequences and efficiently reduce their expression in cancer cells
Source: J Enzyme Inhib Med Chem. 2023 Apr 5;38(1):2198678. doi: 10.1080/14756366.2023.2198678 (PMC10078150; doi:10.1080/14756366.2023.2198678)
Supplement: Supplemental Material [file IENZ_A_2198678_SM1716.pdf]

Supporting Information

Asymmetric triplex metallohelices stabilize DNA G-quadruplexes in promoter oncogene sequences and efficiently reduce their expression in cancer cells

Jaroslav Malina[a], Hana Kostrhunova[a], Hualong Song[b], Peter Scott[b], Viktor Brabec[a]

[a] Czech Academy of Sciences, Institute of Biophysics, Kralovopolska 135, CZ-61200 Brno, Czech Republic
[b] Department of Chemistry, University of Warwick, Coventry, CV4 7AL, UK

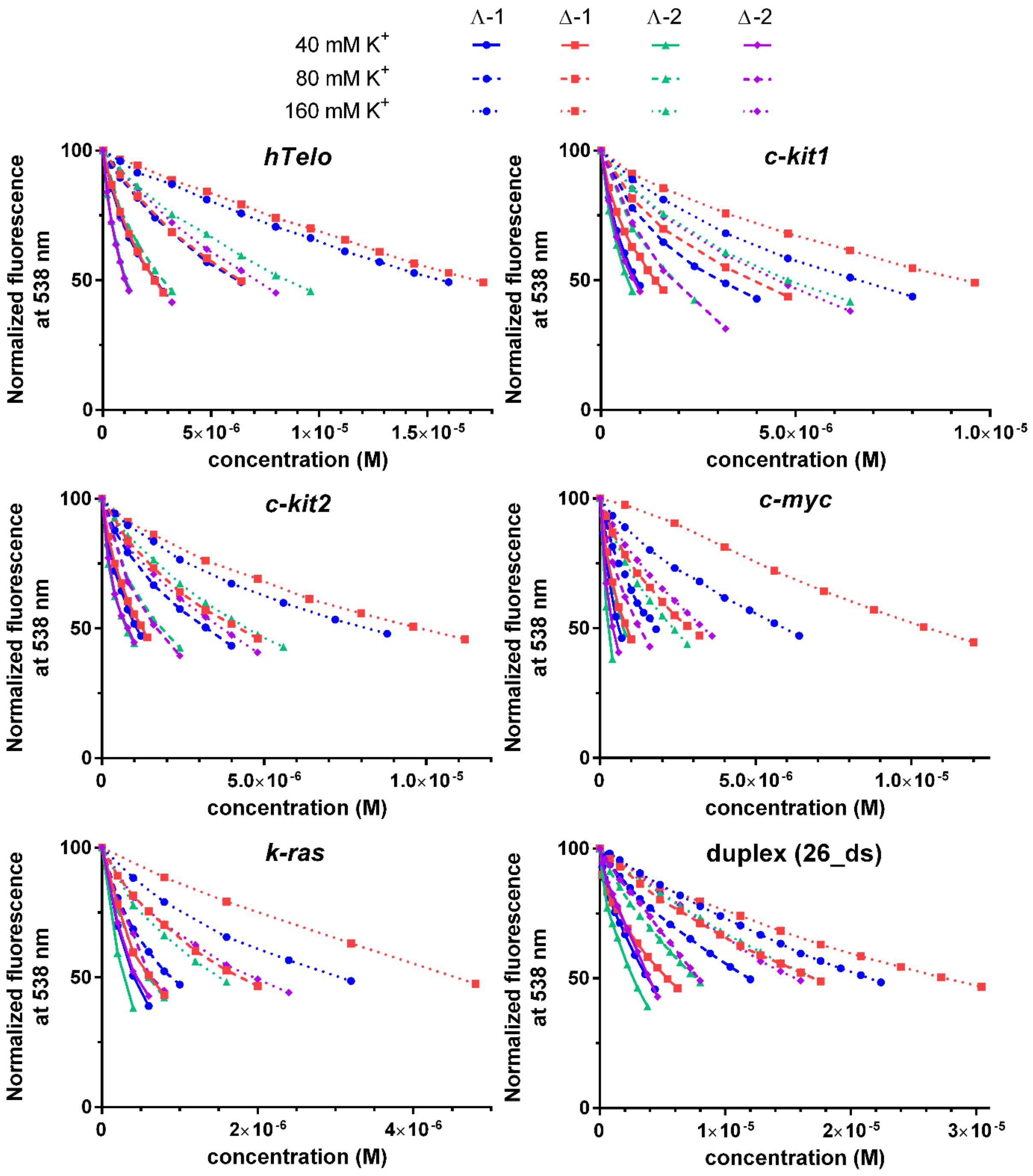

Figure S1. Displacement of thiazole orange from *hTelo*, *c-kit1*, *c-kit2*, *c-myc*, and *k-ras* DNA G4s and from DNA duplex (26_ds) by the enantiomers of **1** and **2** in 10 mM potassium phosphate (pH 7) and various concentrations of KCl.

Table S1. DC_{50} values (μM) for 22_*hTelo*, 22_*c-myc*, 22_*c-kit1*, 21_*c-kit2*, 22_ *k-ras*, and 26_ds in 40 mM K^+, 30 mM KCl and 10 mM potassium phosphate buffer (pH 7), determined by FID upon addition of metallohelices.

Compound	TO displacement (DC_{50})					
	hTelo	***c-kit1***	***c-kit2***	***c-myc***	***k-ras***	**duplex**
**Λ-1**	2.2 ± 0.1	0.96 ± 0.04	1.05 ± 0.04	0.53 ± 0.02	0.42 ± 0.02	3.8 ± 0.2
**Δ-1**	1.95 ± 0.09	1.41 ± 0.03	1.26 ± 0.06	0.80 ± 0.04	0.65 ± 0.03	5.3 ± 0.3
**Λ-2**	1.05 ± 0.05	0.79 ± 0.03	0.78 ± 0.05	0.30 ± 0.03	0.27 ± 0.01	2.6 ± 0.1
**Δ-2**	1.06 ± 0.03	0.87 ± 0.02	0.85 ± 0.05	0.40 ± 0.03	0.45 ± 0.02	3.9 ± 0.2

Table S2. Selectivities of metallohelices towards 22_*hTelo*, 22_*c-myc*, 22_*c-kit1*, 21_*c-kit2*, and 22_ *k-ras* in 40 mM K^+, 30 mM KCl and 10 mM potassium phosphate buffer (pH 7).

Compound	Selectivity (DC_{50})				
	**duplex/ *hTelo***	**duplex/ *c-kit1***	**duplex/ *c-kit2***	**duplex/ *c-myc***	**duplex/ *k-ras***
**Λ-1**	1.7 ± 0.1	4.0 ± 0.3	3.6 ± 0.2	7.2 ± 0.5	9.0 ± 0.6
**Δ-1**	2.7 ± 0.2	3.6 ± 0.2	4.2 ± 0.3	6.6 ± 0.5	8.2 ± 0.6
**Λ-2**	2.5 ± 0.1	3.3 ± 0.2	3.3 ± 0.2	8.7 ± 0.9	9.6 ± 0.5
**Δ-2**	3.7 ± 0.2	4.5 ± 0.2	4.6 ± 0.3	9.8 ± 0.9	8.7 ± 0.6

Table S3. DC_{50} values (μM) for 22_*hTelo*, 22_*c-myc*, 22_*c-kit1*, 21_*c-kit2*, 22_ *k-ras*, and 26_ds in 80 mM K^+, 70 mM KCl and 10 mM potassium phosphate buffer (pH 7), determined by FID upon addition of metallohelices.

Compound	TO displacement (DC_{50})					
	hTelo	***c-kit1***	***c-kit2***	***c-myc***	***k-ras***	**duplex**
**Λ-1**	6.2 ± 0.4	3.1 ± 0.2	3.2 ± 0.2	1.89 ± 0.09	0.93 ± 0.06	11.9 ± 0.4
**Δ-1**	6.4 ± 0.3	3.9 ± 0.3	4.2 ± 0.3	3.0 ± 0.1	1.79 ± 0.04	17.0 ± 0.6
**Λ-2**	2.9 ± 0.1	1.93 ± 0.06	2.0 ± 0.1	0.73 ± 0.04	0.58 ± 0.04	7.7 ± 0.3
**Δ-2**	2.4 ± 0.1	1.94 ± 0.08	1.8 ± 0.1	1.23 ± 0.07	0.76 ± 0.08	7.8 ± 0.4

Table S4. Selectivities of metallohelices towards 22_*hTelo*, 22_*c-myc*, 22_*c-kit1*, 21_*c-kit2*, and 22_ *k-ras* in 80 mM K^+, 70 mM KCl and 10 mM potassium phosphate buffer (pH 7).

**Compound**	**Selectivity (DC_{50})**				
	**duplex/ *hTelo***	**duplex/ *c-kit1***	**duplex/ *c-kit2***	**duplex/ *c-myc***	**duplex/ *k-ras***
**Λ-1**	1.9 ± 0.1	3.8 ± 0.3	3.7 ± 0.3	6.3 ± 0.4	12.8 ± 0.9
**Δ-1**	2.7 ± 0.2	4.4 ± 0.4	4.0 ± 0.3	5.7 ± 0.3	9.5 ± 0.4
**Λ-2**	2.7 ± 0.1	4.0 ± 0.2	3.9 ± 0.2	10.5 ± 0.7	13 ± 1
**Δ-2**	3.3 ± 0.2	4.0 ± 0.3	4.3 ± 0.3	6.3 ± 0.5	10 ± 1

Table S5. DC_{50} values (μM) for 22_*hTelo*, 22_*c-myc*, 22_*c-kit1*, 21_*c-kit2*, 22_ *k-ras*, and 26_ds in 160 mM K^+, 150 mM KCl and 10 mM potassium phosphate buffer (pH 7), determined by FID upon addition of metallohelices.

**Compound**	**TO displacement (DC_{50})**					
	hTelo	***c-kit1***	***c-kit2***	***c-myc***	***k-ras***	**duplex**
**Λ-1**	15.7 ± 0.6	6.6 ± 0.4	8.2 ± 0.5	5.9 ± 0.3	3.0 ± 0.2	22.2 ± 0.9
**Δ-1**	17.3 ± 0.7	9.3 ± 0.5	9.9 ± 0.4	10.3 ± 0.4	4.6 ± 0.3	27 ± 1
**Λ-2**	8.5 ± 0.5	4.8 ± 0.3	4.5 ± 0.2	2.3 ± 0.1	1.57 ± 0.06	17.3 ± 0.8
**Δ-2**	7.1 ± 0.4	4.5 ± 0.2	3.7 ± 0.2	3.3 ± 0.2	1.97 ± 0.05	15.9 ± 0.7

Table S6. Selectivities of metallohelices towards 22_*hTelo*, 22_*c-myc*, 22_*c-kit1*, 21_*c-kit2*, and 22_*k-ras* in 160 mM K^+, 150 mM KCl and 10 mM potassium phosphate buffer (pH 7).

**Compound**	**Selectivity (DC_{50})**				
	**duplex/ *hTelo***	**duplex/ *c-kit1***	**duplex/ *c-kit2***	**duplex/ *c-myc***	**duplex/ *k-ras***
**Λ-1**	1.41 ± 0.08	3.4 ± 0.2	2.7 ± 0.2	3.8 ± 0.2	7.4 ± 0.6
**Δ-1**	1.56 ± 0.09	2.9 ± 0.2	2.7 ± 0.2	2.6 ± 0.2	5.9 ± 0.5
**Λ-2**	2.0 ± 0.2	3.6 ± 0.3	3.8 ± 0.2	7.5 ± 0.5	11.0 ± 0.7
**Δ-2**	2.2 ± 0.2	3.5 ± 0.2	4.3 ± 0.3	4.8 ± 0.4	8.1 ± 0.4

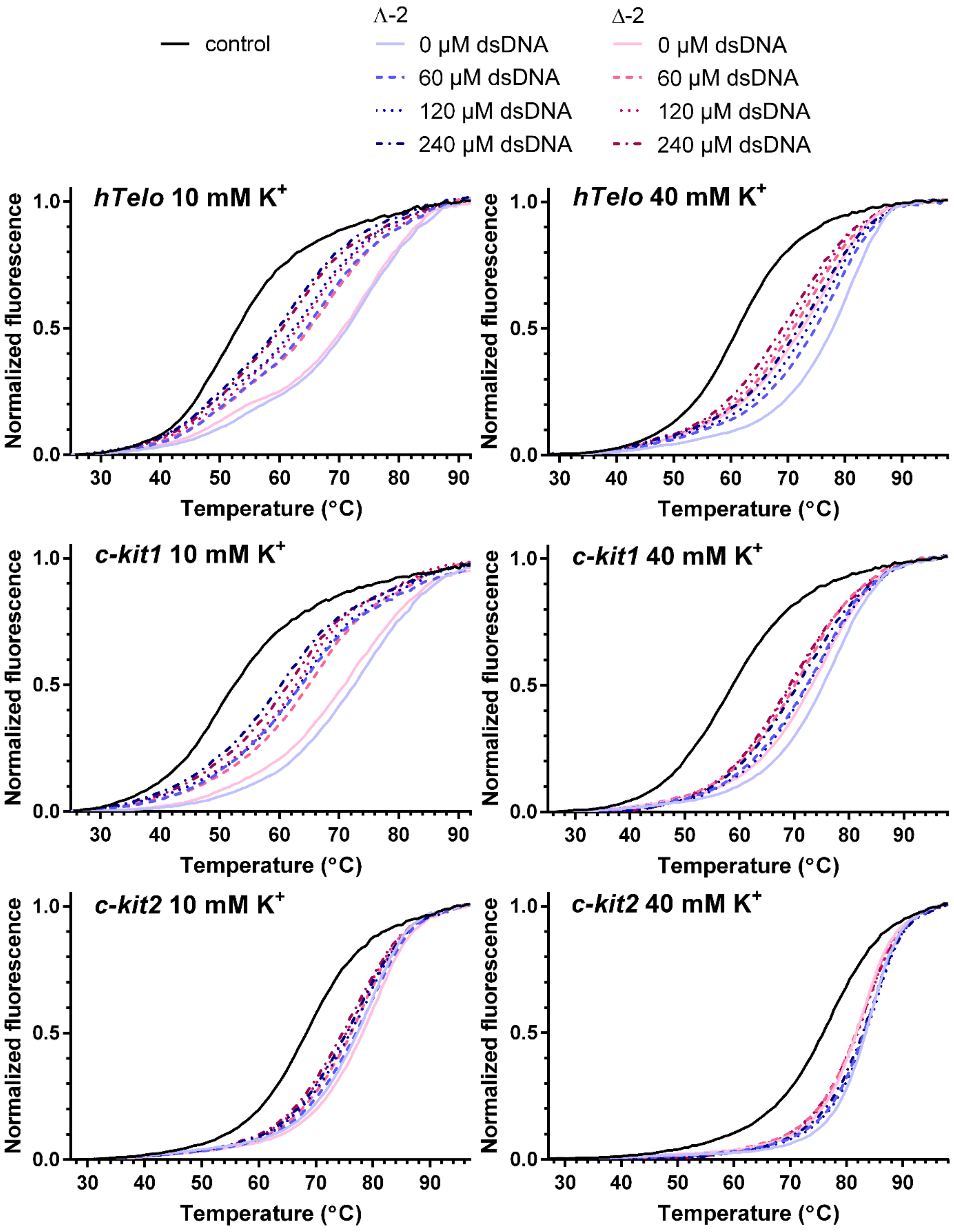

Figure S2. FRET melting curves for *hTelo*, *c-kit1*, and *c-kit2* DNA G4s (0.4 µM) in the absence and in the presence of 0.8 µM enantiomers of **1** and **2** and increasing concentrations (indicated in the figure) of dsDNA. The buffer conditions were 10 mM potassium phosphate (pH 7).

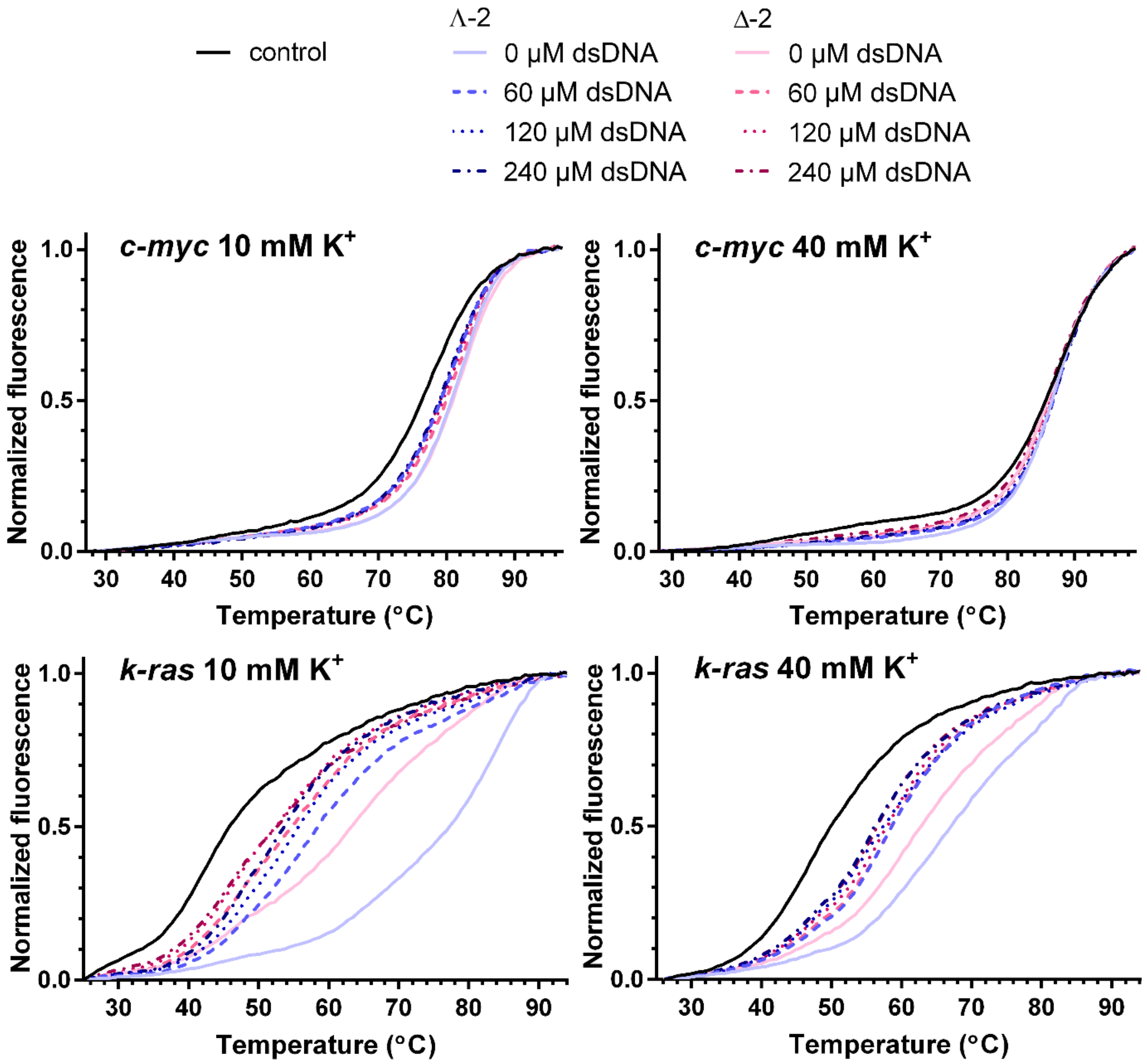

Figure S3. FRET melting curves for *c-myc* and *k-ras* DNA G4s (0.4 μM) in the absence and in the presence of 0.8 μM enantiomers of **1** and **2** and increasing concentrations (indicated in the figure) of dsDNA. The buffer conditions were 10 mM potassium phosphate (pH 7).

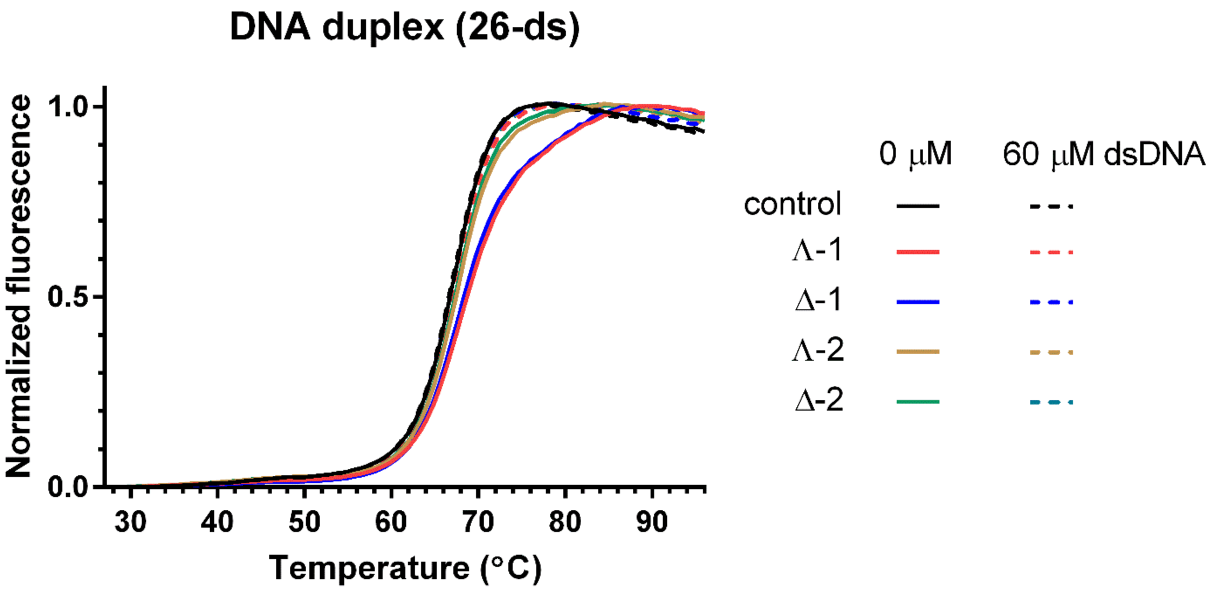

Figure S4. FRET melting curves for the DNA duplex (0.4 μM) in the absence and in the presence of 0.8 μM enantiomers of **1** and **2** and 60 μM dsDNA. The buffer conditions were 10 mM potassium phosphate (pH 7).

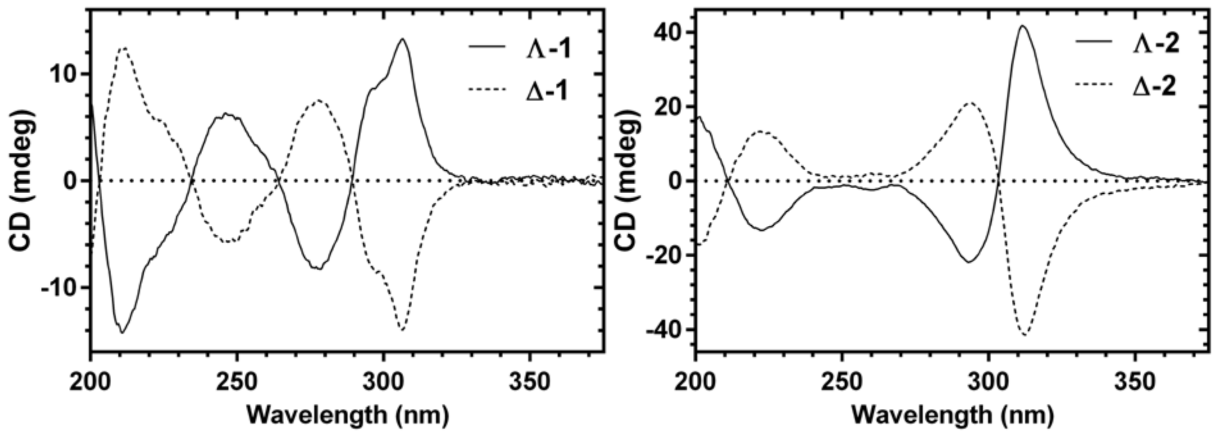

Figure S5. CD spectra of Λ- and Δ-enantiomers of **1** and **2** (5 μM) in 10 mM potassium phosphate (pH 7)

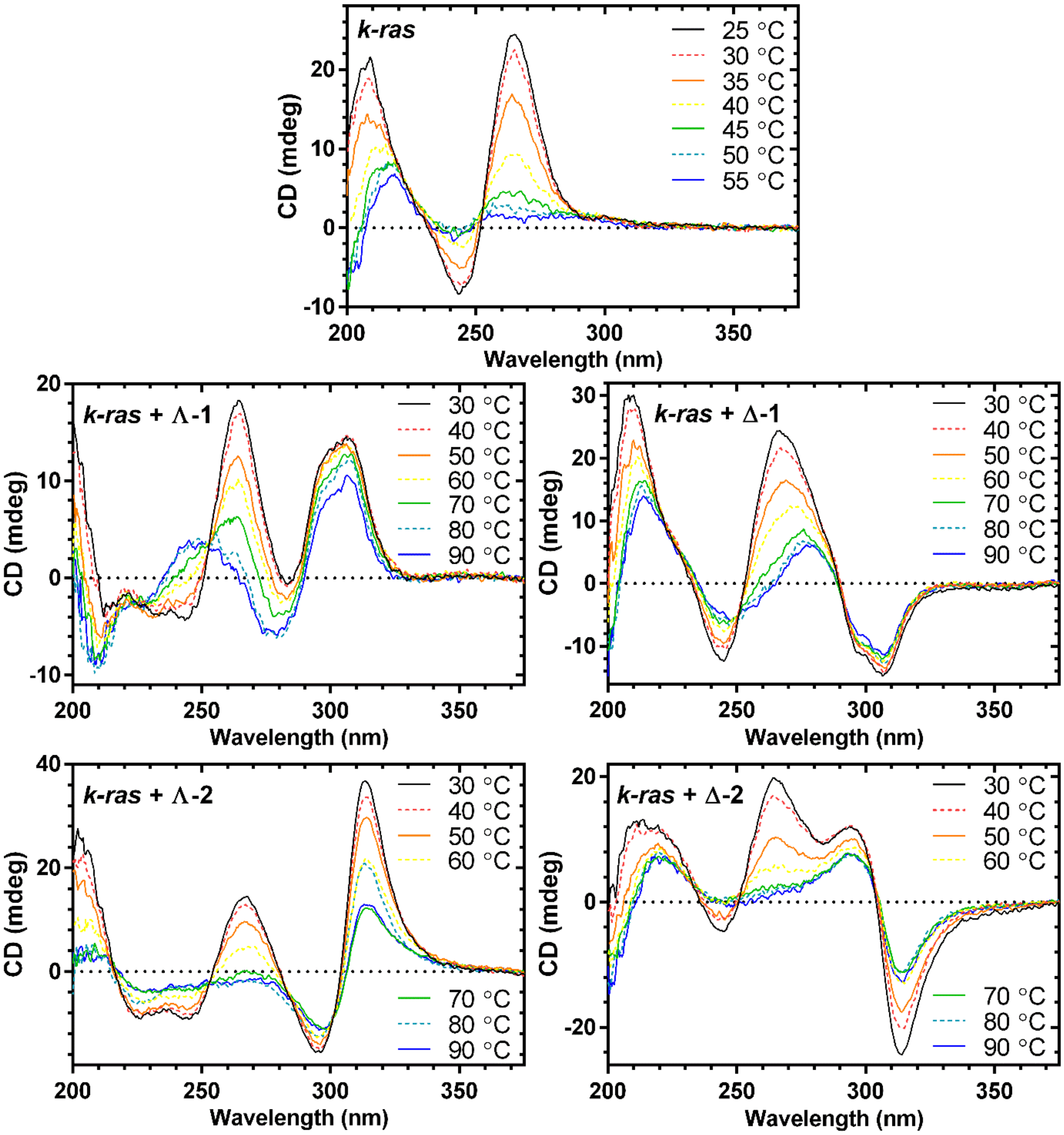

Figure S6. CD spectra of *k-ras* G4 (2.5 µM) in the absence and in the presence of Λ- and Δ-enantiomers of **1** and **2** (5 µM) at varying temperatures (average of two scans). The buffer conditions were 10 mM potassium phosphate (pH 7).

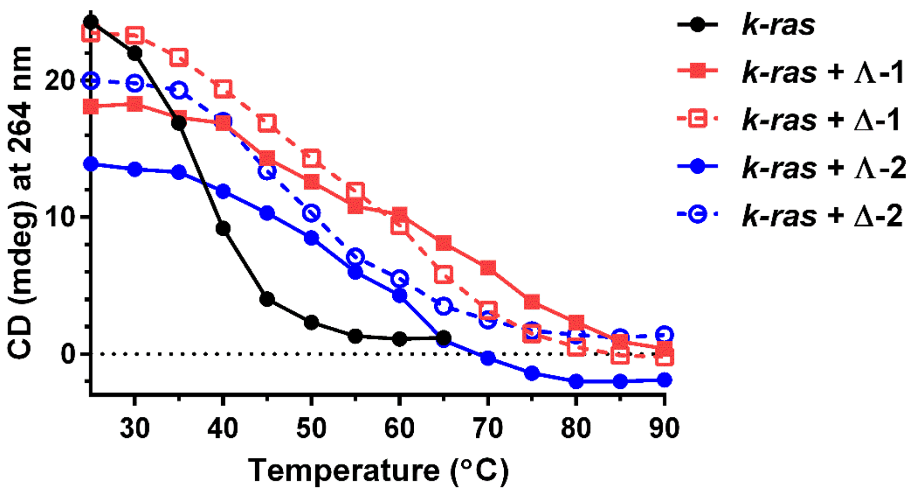

Figure S7. Changes in CD signals at 264 nm as a function of temperature.

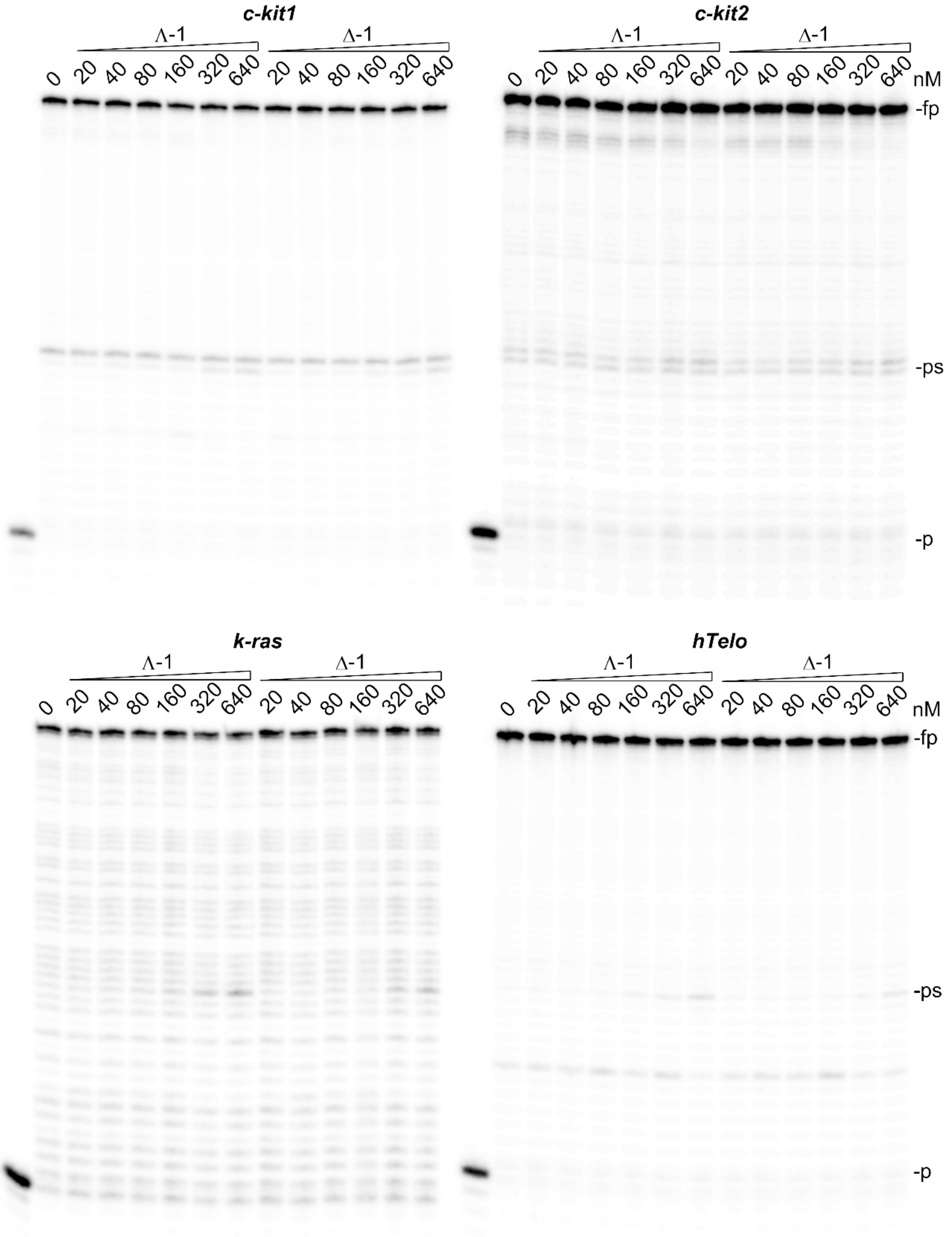

Figure S8. Autoradiograms of 12% PAA sequencing gels with products of DNA synthesis on the templates containing *c-kit1*, *c-kit2*, *k-ras*, and *hTelo* G4-forming sequences in the presence of increasing concentrations of **Λ-1** and **Δ-1**. *fp*, *ps*, and *p* correspond to full-length product, pausing site by G4, and primer, respectively.

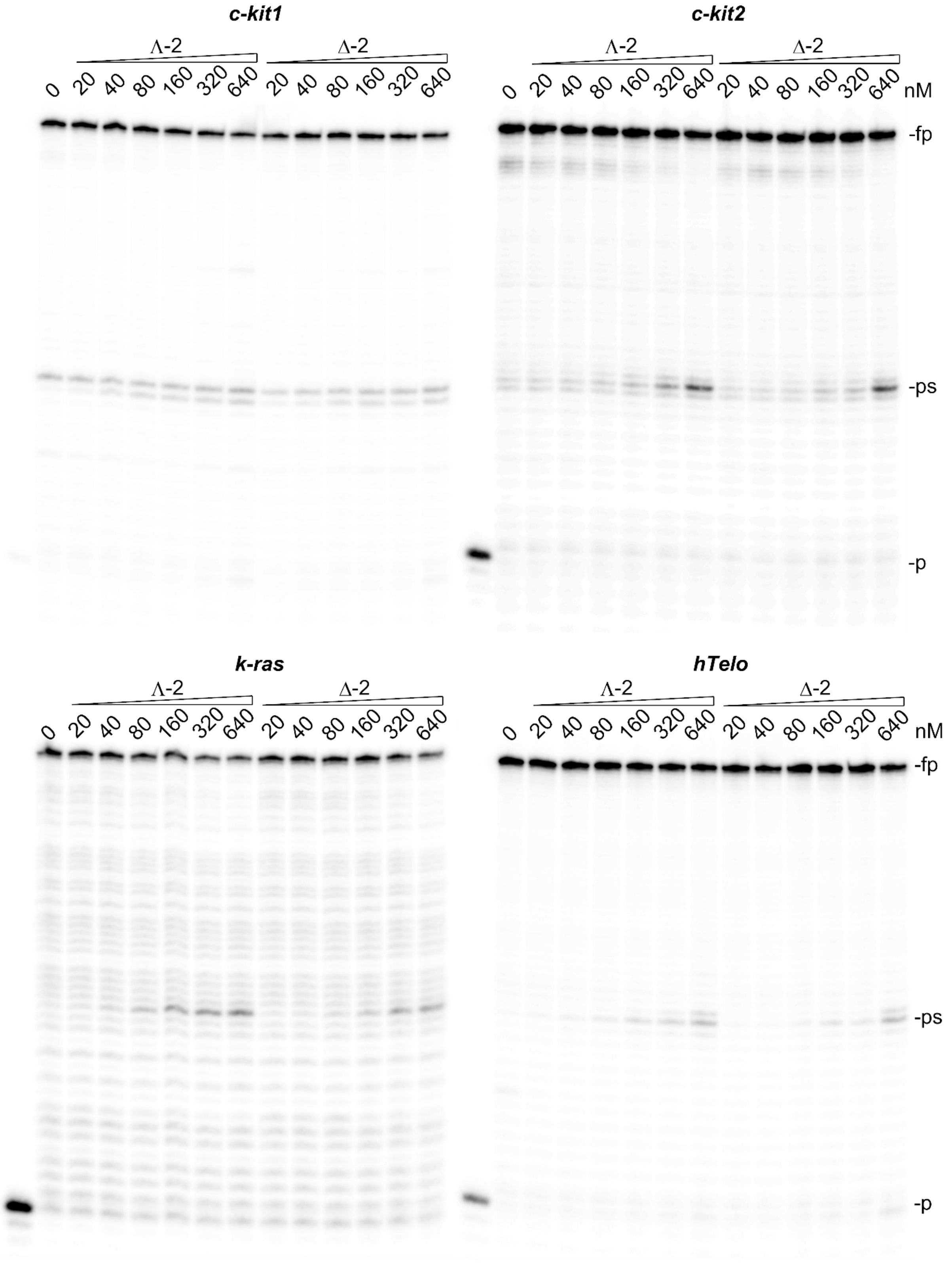

Figure S9. Autoradiograms of 12% PAA sequencing gels with products of DNA synthesis on the templates containing *c-kit1*, *c-kit2*, *k-ras*, and *hTelo* G4-forming sequences in the presence of increasing concentrations of **Λ-2** and **Δ-2**. *fp*, *ps*, and *p* correspond to full-length product, pausing site by G4, and primer, respectively.

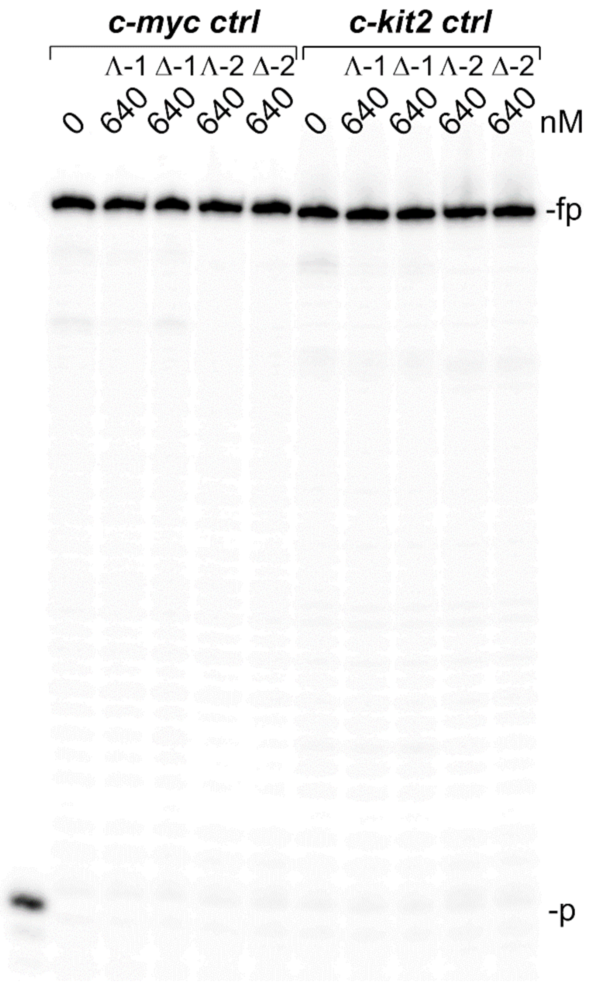

Figure S10. **A**utoradiogram of the 12% PAA sequencing gel with products of *Taq* polymerase DNA synthesis conducted at 55 ºC across *c-myc* and *c-kit2* control templates (30 nM, unable to form G4s) in the presence of 640 nM metallohelices. *fp* and *p* correspond to full-length product and primer, respectively.

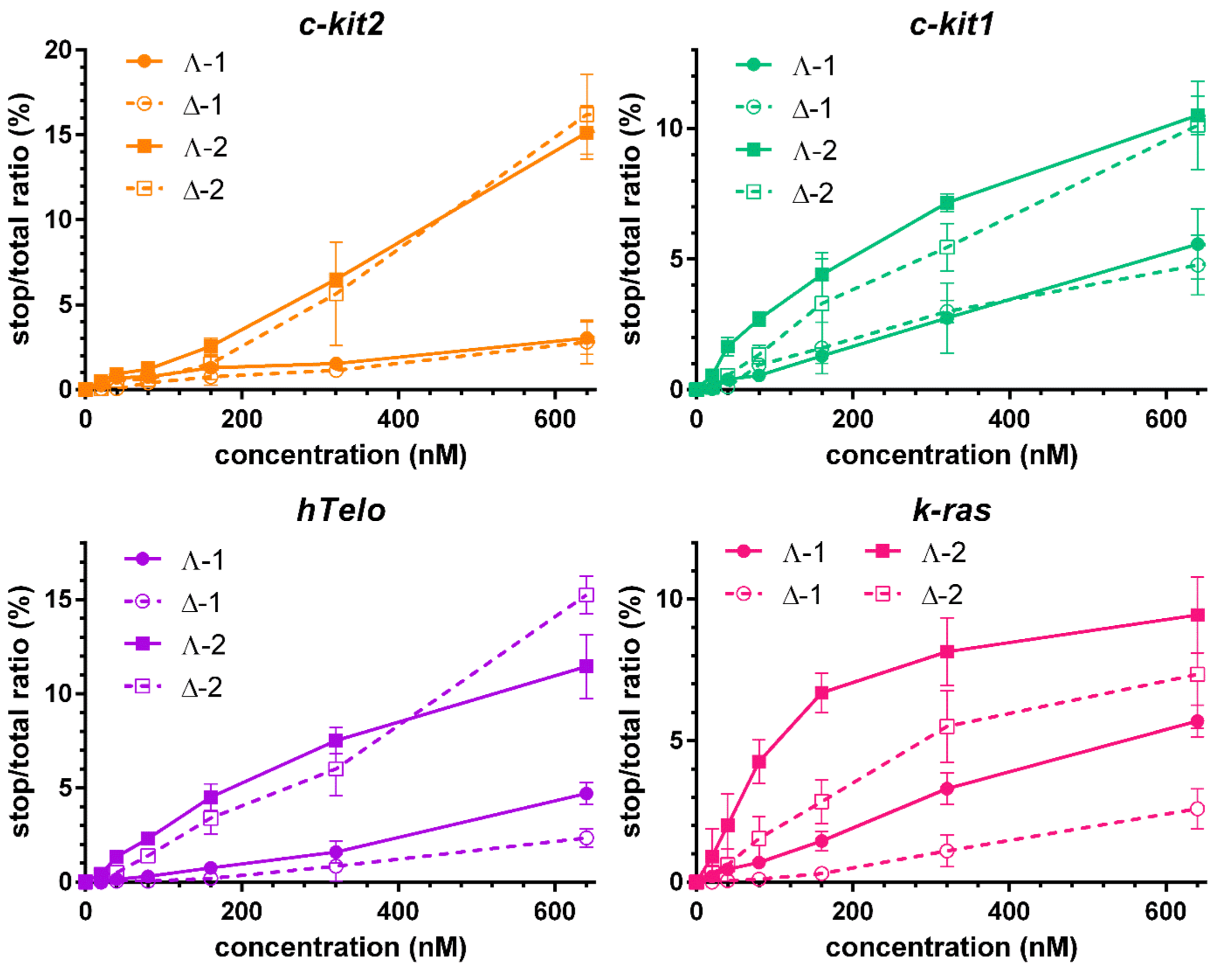

Figure S11. Inhibition of DNA synthesis across templates containing *c-kit2*, *c-kit1*, *hTelo*, and *k-ras* G4-forming sequences. Plots showing the ratio of the radiation corresponding to pausing sites to total radiation of the lane vs. the concentration of **1** and **2** enantiomers. The results are expressed as mean ± SD from two independent experiments.
